# Out of the Loop, in Your Bubble: Mind Wandering Is Independent From Automation Reliability, but Influences Task Engagement

**DOI:** 10.3389/fnhum.2018.00383

**Published:** 2018-09-20

**Authors:** Jonas Gouraud, Arnaud Delorme, Bruno Berberian

**Affiliations:** ^1^Cognitive Engineering and Applied Neuroscience Unit, Office National d’Etudes et de Recherches Aérospatiales, Salon-de-Provence, France; ^2^Centre de Recherche Cerveau & Cognition – UMR5549 (CerCo), Toulouse, France; ^3^Swartz Center for Computational Neuroscience, University of California, San Diego, San Diego, CA, United States

**Keywords:** mind wandering, perceptual decoupling, out-of-the-loop, complacency, automation, reliability, trust, mental demand

## Abstract

This study examined the influence of automation reliability on task-unrelated mind wandering (MW) frequency and the impact of MW on task engagement. Automated environment features make it particularly prone to increase MW frequency. Through mechanisms like complacency or agency, automating a task could increase MW frequency for the operator. For safety-critical industries, the lower perception and degraded stimuli processing associated with MW, summarized by the term “decoupling hypothesis,” are particularly concerning. Sixteen participants supervised an autopilot avoiding obstacles with two levels of reliability. Each condition lasted 45 min. We recorded thoughts as either pertaining to being focused, task-related MW or task-unrelated MW. We also recorded perceived mental demand, trust regarding the autopilot and oculometric measures. Based on questionnaire results, our protocol succeeded in inducing more mental demand and lower trust when the automation was unreliable. Attentional states were not correlated, nor did it influence trust in the system reliability. On the contrary, mental demand ratings and pupil diameter were lower during both task-related and task-unrelated MW, compared to those during the focus attentional state. This shows that perceptual decoupling also affects the engagement of operators in automated environments, which may dramatically lower their ability to supervise automation efficiently. This research informs human-automation designers to consider operator engagement when creating automated systems.

## Introduction

Automation has fundamentally changed our working environments. Particularly, the industry makes extensive use of automated systems to design more reliable and efficient environments. However, side-effects of these automated systems have been observed. Particularly, the externally imposed task of maintaining sustained attention of human operators focused for long periods of time on the automated system – which has a very low probability of failure – causes progressive vigilance decrement preventing efficient automation supervision (Amalberti, 1999). The task of detecting and reacting to alerts, which seldom occur and are drowned in noise, has become stressful and increasingly difficult (Mackworth, 1948; Hancock, 2013). Problems linked to moving operators to an automated system supervising role have been summarized by the term ‘out-of-the-loop (OOTL) performance problem’ (Endsley and Kiris, 1995). The OOTL problem has been studied in laboratories (Baxter et al., 2007; Louw et al., 2015), but it is still difficult to quantify it after decades of research (Baxter et al., 2012). One of the key components of OOTL is often linked to decreased vigilance leading to insufficient environment information extraction (Pattyn et al., 2008). At an operational level, OOTL is regularly encountered among the causes for various incidents and accidents (Federal Aviation Authority, 1972, 1995; Bureau d’Enquête et d’Analyse, 2013).

Mind wandering (MW) may play an important role in this context. MW is a family of experiences related to the human mind’s tendency to engage in thoughts away from the ‘here and now’ (Smallwood and Schooler, 2006). We all mind wander to some extent in our daily lives (Killingsworth and Gilbert, 2010). We sometimes do it willingly to evade a boring environment, but it can also happen without us being able to control it, or even being conscious that our mind had wandered (Smallwood, 2013; Golchert et al., 2016; Seli et al., 2016). MW may start with some thoughts related to the task (therefore called task-related MW, as when thinking about task performance) or be completely unrelated to the task (called task-unrelated MW, as when thinking about dinner while driving). It is important to note that MW in general is thought to have some evolutionary use, possibly helping to solve problems or lower cognitive fatigue (Mooneyham and Schooler, 2013; Gouraud et al., 2017a). More particularly, task-related MW could allow future planning (Schooler et al., 2014). Task-unrelated MW is more likely to occur in monotonous environments (Eastwood et al., 2012), or when operators perform familiar (Bastian et al., 2017) or long tasks (Smallwood and Schooler, 2015). Interestingly, we have recently shown that an automated context could increase MW frequency (see Gouraud et al., 2017b for preliminary results): we demonstrated in a plane simulator that operators cannot always control their MW. Understanding how automation influences MW and how MW influences operators’ engagement is of high interest for safety-critical industries like aeronautics, nuclear plants or automobiles. A first issue concerns the automation features causing an increase in MW frequency. As previously stated, automated environments are generally repetitive and monotonous, with very few target events, all of which are characteristics known to increase MW. However, they are not the only features of automation that could influence MW. Among the most important characteristics of automated systems, reliability is considered as one of the causes of the observed vigilance decrement observed in OOTL episodes (Metzger and Parasuraman, 2001). The paradox of ultra-safe systems is that the absence of any failure for a prolonged period of time will lead operators to make commission errors – i.e., accept an automation recommendation despite the fact that it may be wrong (Amalberti, 2001). This phenomenon is called automation-induced complacency (Parasuraman et al., 1993). Complacency is the adoption of a non-optimal information sampling behavior based on over-trust in the system’s capabilities due to a minimization of the automation failure probability (Moray and Inagaki, 2000; Innes-Jones and Scandpower, 2012). Even though it can emerge unconsciously, complacency can be seen as a multiple-task strategy to optimize the global output when supervising an automated system, while also performing a more engaging task. However, this strategy can sometimes lead to dramatic failures in safety critical environments. Multiple meta-studies reported complacency as being one of the main reasons for an important number of crashes (Wiener, 1981; Parasuraman and Riley, 1997; Funk et al., 1999). Complacency may lead operators to disengage from the task and reallocate their cognitive resources to more personal matters, increasing MW frequency. MW frequency would therefore increase with automation reliability.

A second issue concerns the impact of MW on safety. One of the most threatening aspect of MW for safety is the decoupling from the environment (Schooler et al., 2011). Operators engaged in an episode of MW will experience a deterioration of their encoding of external information. MW disrupts visual information flow by reducing pupil diameter (Smallwood et al., 2011; McIntire et al., 2014) and increasing blink frequency (Smilek et al., 2010). Neuronal studies demonstrated an increase of alpha wave power during MW, linked with sensory suppression (O’Connell et al., 2009; Foxe and Snyder, 2011), and a reduction of Event Related Potentials linked to external information perception and processing (Smallwood et al., 2008). At the behavioral level, this decoupling translates into a decrease in performance. Reaction exhibits higher variability (Bastian and Sackur, 2013), while omissions and anticipations are more common (Cheyne et al., 2011). Accuracy was shown to decrease in both simple paradigms (Kam et al., 2012) and more ecological ones (Yanko and Spalek, 2014). This evidence demonstrates that MW disrupts online adjustment of behavior (Kam et al., 2012), despite recent criticism of some of the paradigms used (Head and Helton, 2016). Particularly, MW-induced decoupling might lead supervisors to disengage from the task and overlook some failures, leading to OOTL problems. Such disengagement should be observed both at the behavioral and physiological levels.

Even though multiple studies have investigated MW-induced perceptual decoupling, no attempt has been made, to our knowledge, to do so when supervising automation. We report in this article an experiment on the evolution and consequences of MW within an automated environment of varying reliability. Our hypotheses are that (1) higher reliability increases task-unrelated MW by creating complacency and (2) MW-induced decoupling impacts operators’ engagement (perceived mental demand and oculometric signal) under operational conditions.

## Materials and Methods

### Participants

Sixteen participants (3 female) performed the experiment (age ranging from 22 to 43 years old; *M* = 29.0, *SD* = 5.8). The participants enrolled in this study were volunteers from our company (ONERA, the French Aerospace Lab). All participants had normal or corrected-to-normal visual acuity. All participants were unfamiliar with the concepts at hand and the LIPS environment. All participants signed a written declaration of informed consent. The procedure was approved by ONERA and conducted in accordance with the World Medical Association Declaration of Helsinki.

### Task

#### Environment

**FIGURE 1:**
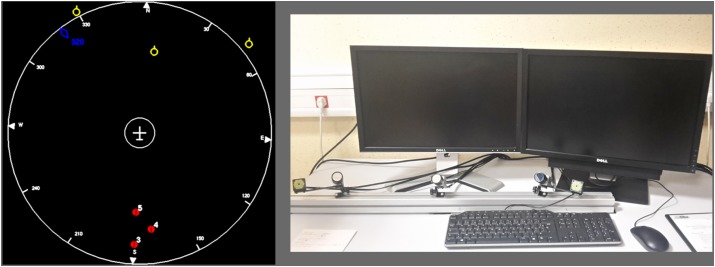
Screenshot of the LIPS interface and the environment. On the right, the general set-up of the experiment is depicted. One of the screens is used for the task and the other is used for questionnaire probes. On the left, the screen used for the task is focused on. The plane in the center is static and the surrounding objects (yellow and red numbered symbols) are moving. During left and right avoidance maneuvers, the plane is again static and the background is rotated.

We used the LIPS (*Laboratoire d’Interactions Pilote – Système*, or Pilot-System Interaction Laboratory) environment developed at ONERA to program our experiment (see **Figure 1**). An unmanned air vehicle (UAV), depicted as a plane seen from above, stayed at the center of a 22-inch 2D radar screen and moved following waypoints arranged in a semi-straight line. Clusters of obstacles appeared along the way (every 45 s on average). Each cluster could contain between 1 to 5 obstacles, including one on the trajectory. When an obstacle was present on the trajectory (this situation is called “conflict”), the autopilot detected it and initiated a deviation automatically. This avoidance trajectory could result in a left or right turn, depending on the placement of all obstacles in the conflict. The participants were instructed to monitor the UAV and correct any mistake that the autopilot may make (i.e., an avoidance trajectory that would result in an impact with another obstacle). When the autopilot decided that the obstacle was not on the trajectory anymore, it initiated a change in the avoidance trajectory to head to the next checkpoint. The LIPS environment includes a physics engine to reproduce convincing *Rafale* military aircraft motion behavior. The LIPS was displayed on the left screen within the environment shown in **Figure 1**.

#### Conditions

Participants were required to monitor the autopilot avoiding obstacles. They had to click on an “*Acquittement*” (acknowledgment) button to acknowledge automated avoidance decisions as soon as they saw it (twice per trial, once to acknowledge avoidance of the object and once to acknowledge the return to normal trajectory after avoiding the object). A feedback message was displayed to the participants. Finally, if participants detected an incoming collision warning, they were instructed to click on the button “*Changement d’altitude*” (change height) so that the UAV would perform an emergency descent to avoid colliding with the obstacle. A feedback message was displayed in that case as well. Collisions could occur during the avoidance trajectory, if there was another obstacle on the bypass trajectory chosen by the autopilot. In that case, an orange circle appeared around the obstacle to indicate that the UAV was too close to some object, threatening the safety of the flight. Two conditions were proposed. Under the “Risky” condition, the autopilot made an error (choosing the wrong side) leading to a collision in 40% of the trials (27 errors in total), selected randomly. This number was chosen so that there would be a significant number of collisions, while keeping the automated system performance above the chance expectation (50%). Under the other “Safe” condition, the autopilot only made 5 errors (7% errors; errors on trials 24, 40, 56, 62 and 64). Each condition contained 67 clusters of obstacles. All decisions and collisions were predefined and, therefore, they were the same for all subjects.

#### Experience Sampling Probes

**FIGURE 2:**
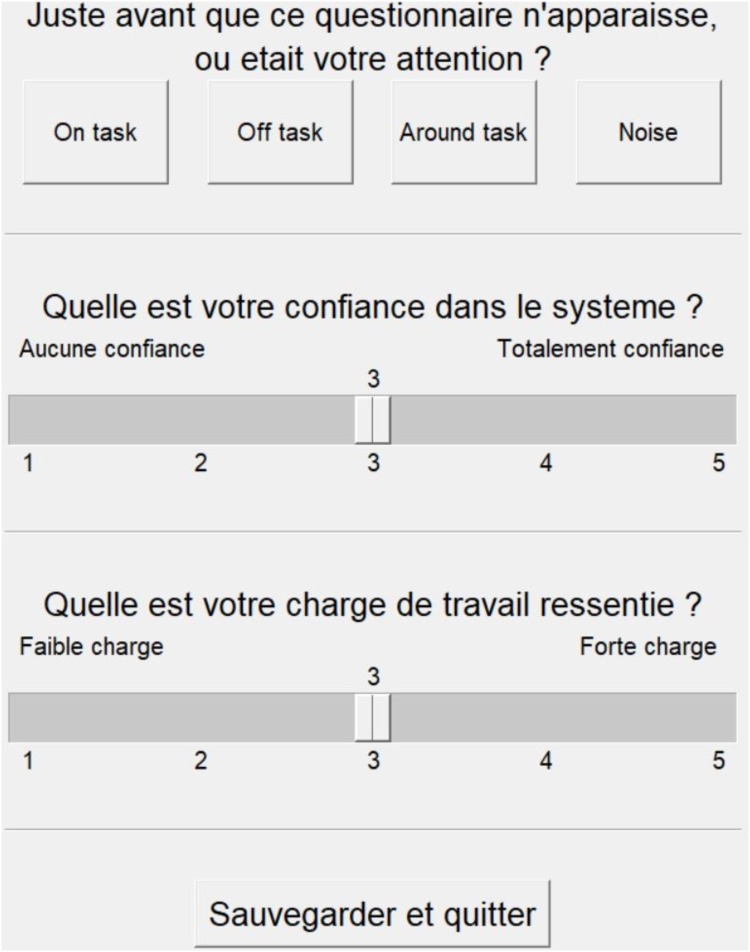
Screenshot of the experience-sampling probes in French.

Python 3.6 was used to program experience sampling probes. On average every 2 min, an experience sampling probe appeared on a secondary 10-inch screen next to the main screen. For technical reasons, the obstacle-avoidance task was not paused when the experience sampling probes appeared. Participants were asked to enter it as soon as it appeared, and any successful or failed trial during this interval would not be taken into account to compute their performances. Participants were informed that the questionnaire probes were for informational purposes only and were not used to assess performance. This limited the possibility of participants being reluctant to report their distraction. Participants were required to answer the following questions (originally in French, see **Figure 2**): “When this questionnaire appeared, where was your attention directed?” Answers could be “On the task” (focused, e.g., thinking about the next obstacle, the decision to make, the incoming waypoint), “Something related to the task” (task-related MW, e.g., thinking about performance, interface items, last trial), “Something unrelated to the task” (task-unrelated MW, e.g., thinking about a memory, their last meal, or a body sensation) or “External distraction” (e.g., conversation, noise). The preceding examples were given to participants to illustrate each category prior to the experiment. We were primarily interested in reports of being focused or having task-related or task-unrelated MW. The possibility of reporting “task-related MW” was proposed to avoid participants reporting task-unrelated MW when thinking about their performance (Head and Helton, 2016). The answer “Noise” was proposed to avoid participants reporting MW if they were focused on any external signal. The second question was “how much do you trust the system?” We made explicit that this was the trust in the ability of the system to perform its task without errors. Answers ranged from “no trust” to “total trust” on a 5-point Likert scale. Finally, the third question was: “what is your perceived workload?” We used the word “workload” (“*charge de travail*” in French) because the term is generally understood by everyone. Perceived mental demand was measured as an important aspect of task engagement (Parasuraman and Riley, 1997). However, we further clarified this term for the participant prior to starting the experiment as the perceived quantity of mental effort needed to achieve the objectives. We refer to this question as “perceived mental demand” throughout the rest of the paper. Answers ranged from “low mental demand” to “high mental demand” on a 5-point Likert scale.

#### Procedure

**FIGURE 3:**
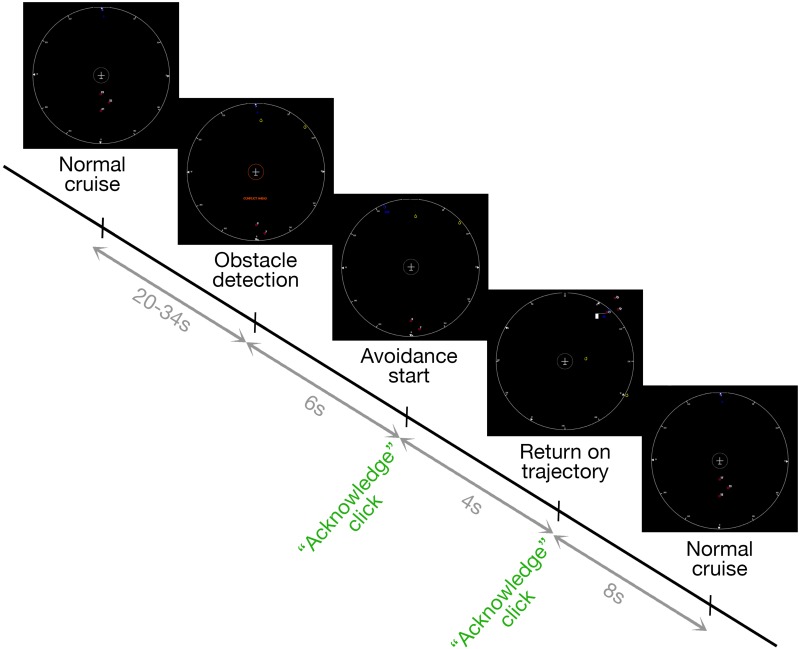
Step by step explanation of a trial. The UAV moves forward cruising without events for 27 s on average. The automated pilot will detect any obstacle along the way and decide which way to go (left or right). Once it decides the direction, participants must click on “Acknowledge.” When the automated pilot decides that the obstacle is not on the trajectory anymore, it heads to the next checkpoint and participants must once again click on “Acknowledge.” However, when the automated pilot chooses the wrong side, participants must click on “Altitude Change” to avoid the collision. At any randomly selected moment, an experience-sampling probe may appear.

Participants were explicitly instructed that detection accuracy was more important than speed of response. Each participant performed under the two conditions on two separate days in a counterbalanced way. Each day started with an explanation of the task, followed by a 10-min training period and a 50-min session under the proper condition. Each session contained 67 clusters of obstacles, totaling 201 obstacles. Each cluster contained between 1 and 5 obstacles, including one on the trajectory. Clusters were separated by 45 s on average. 20 probes were responded to under each condition (see **Figure 3**). The distribution of the experience-sampling probes was not correlated with events on the obstacle-avoidance task, in order to minimize performance influence over experience-sampling reports (Head and Helton, 2016). The “Risky” condition included six conflicts with a probe presented within the 10-s interval following the conflict, while the “Safe” condition included seven conflicts with a probe presented within the 10-s interval following the conflict.

### Data Collection

The raw data supporting the conclusions of this manuscript will be made available by the authors, without undue reservation, to any qualified researcher.

#### Experience-Sampling Probes

Comma Separated Value (CSV) text files were used to store all answers from each session with each subject. The exact appearance time was saved along with each answer, in order to synchronize the questionnaire data with the oculometric signal.

#### Oculometry

Oculometric data was recorded using the SmartEye Pro 3.0 hardware and the SmartEye 7.1.0 software. The system included two infrared illuminators and three cameras (120 Hz sampling frequency) placed below the computer screen (see **Figure 1**). Gaze calibration was performed using a 4-point grid, using the Gaze Calibration Client proposed by SmartEye. The SmartEye software gave an average of both pupil diameters. When only one eye was available, then this pupil was used and the software indicated degraded quality of measure.

#### Performance

We recorded button clicks throughout both conditions. Each button click was saved along with its timestamp in a CSV text file by the LIPS environment.

### Data Analysis

#### Experience Sampling Probes

We used R-Studio 1.0.143 and R 3.4.1 (RStudio Team, 2015; R Core Team, 2016) to analyze the data.

#### Oculometry

We defined oculometric epochs as data during the 10-s intervals preceding each questionnaire. This duration is in line with the literature (He et al., 2011; Franklin et al., 2013; Bixler and D’Mello, 2014, 2015). We performed pupillometry filtering and processing using the R packages *reshape* (Wickham, 2007), *psych* (Revelle, 2017), *ggplot2* (Wickham, 2009, 2), and *robfilter* (Fried et al., 2014). We only seized the pupil diameter when the subject was looking at the main screen to avoid any luminosity effect (e.g., to avoid reporting effects when it was only a case of the people reporting MW looking more outside the screen). Pupil diameters smaller than 1 mm and larger than 10 mm were excluded (due to the physical limits of pupil diameter, see Lemercier, 2014). Pupil diameters differing from the preceding value by more than 80% were also excluded (due to pupil dynamic limits). Pupil diameter with a quality metric (computed by the SmartEye software) below 0.01 were excluded, in order to discard tracking losses (given by a quality of 0). 10-s epochs were discarded if their resulting pupil diameter series consisted of more than 30% discarded samples. We excluded 5.5% of all segments, which is in line with the literature (Smallwood et al., 2011). Resulting segments were completed using linear interpolation if necessary. After interpolation, a second moving average filter was applied (moving window of 50 frames or 417 ms). We also discarded all epochs that included some actions by participants during the interval (i.e., if participants clicked on a button during the 10 s). This ensured that all epochs were free of phasic activity linked to decisions (which could mask the MW influence). Finally, the data for each participant were standardized by subtracting the mean and dividing by the standard deviation of all retained epochs for this participant.

Fixations, saccades and blinks were computed by the SmartEye Pro software. Blinks were computed using 700 ms sliding windows. Saccades were defined in SmartEye Pro parameters as gaze velocity over 35 deg/s. Saccades were limited to 200 ms. Fixations were frames associated with a gaze velocity below 15 deg/s.

#### Performance

Performance was assessed by determining if participants clicked when they were required to do so. “Acknowledge” command for the beginning of the avoidance trajectory was considered missed if participants did not click before the UAV starts the return on trajectory (4 s delay, see **Figure 3**). “Acknowledge” command for the return on trajectory was considered missed if participants did not click at most 10 s after the start of the return on trajectory. “Altitude change” command was considered missed if the participant did not click on it during the 15 s preceding the collision.

## Results

### Mind Wandering Frequency

We split the 50-min sessions into five blocks of 10 min containing five experience-sampling probes each. Participants reported on average 1.38 task-related MW episodes (*SD* = 1.14) and 1.80 task-unrelated MW episodes (*SD* = 1.52) per block. This rate is consistent with previous studies (Smallwood and Schooler, 2006, 2015; Kam et al., 2011; Gouraud et al., 2017b). Each participant reported on average 0.79 external distractions (*SD* = 1.21) during each session. Given that this represented only 3% of all reports, we approximated attentional state as a ternary state – i.e., as being either in focused, task-related MW and task-unrelated MW states.

We investigated the first hypothesis (influence of trust over MW rates) by looking at task-related and task-unrelated MW frequency evolution over time and conditions (see **Table 1** for a description). We used the *lme* function (Pinheiro et al., 2017) and the *anova* function (R Core Team, 2016) to perform a linear nested mixed-effect analysis including a different number of reports between attentional states (Winter, 2013). We considered Blocks as a categorical variable. We defined a random intercept for subjects to take our repeated-measure design into account. No random slope were possible because of convergence problems due to not having enough data. Visual inspection of residual plots did not reveal any obvious deviations from normality or homoscedasticity. Each model, starting from the baseline without any predictor, added one predictor or interaction to the preceding model, until the complete model was reached. *P*-values were obtained by likelihood ratio tests using ANOVA on nested models. All results are gathered in **Table 2**, bold values being significant.

**Table 1:** Descriptive of MW frequency per block (in percentage of reports in one block).

	Task-related MW		Task-unrelated MW	
Block	Mean	*SD*	Mean	*SD*
Block 1	32.8	23.5	30.2	29.6
Block 2	26.4	23.5	31.2	30.4
Block 3	22.2	20.0	33.4	31.7
Block 4	23.9	20.6	37.6	25.2
Block 5	33.1	25.3	47.3	33.0

**Table 2:** Summary of statistics regarding the influence of blocks and condition over task-related and unrelated MW frequency.

	Task-related MW		Task-unrelated MW	
Effect added	*df*	χ^2^	*p*-value	χ^2^	*p*-value
Block	4	8.71	0.069	**14.50**	**0.006**
Condition	5	0.69	0.406	0.42	0.518
Block:Condition	9	**12.28**	0.015	4.57	0.358

*All values in bold are significant at p < 0.05.*

**FIGURE 4:**
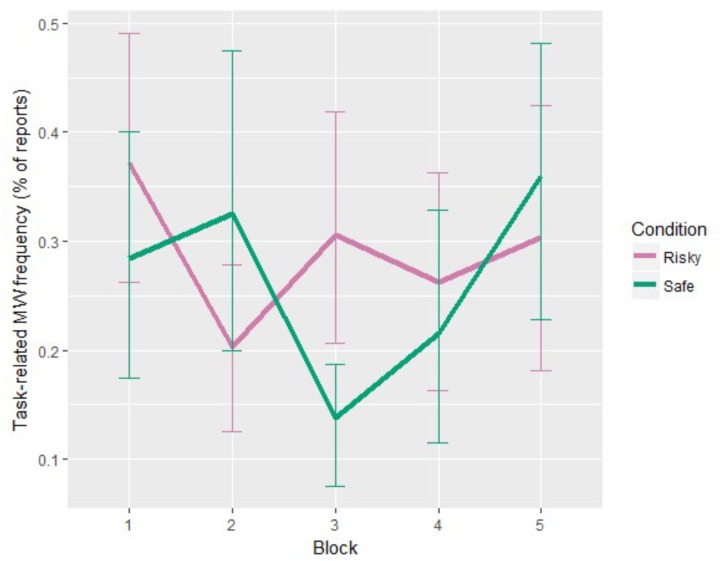
Task-related MW frequency evolution for each condition (error bars show the 95% confidence intervals based on bootstrap).

Blocks did not significantly influence task-related MW (see **Figure 4**). There was a significant interaction between blocks and conditions, χ^2^ = 12.28, *p* = 0.015. Without specific *a priori* predictions regarding the block-by-block evolution, we used the *glht* (Hothorn et al., 2017) and *mes* (Del Re, 2014) functions of *R* to conduct Tukey’s *post hoc* tests on the model, including Block and Condition interaction. Tests revealed that task-related MW frequencies were significantly higher under the “Risky” condition during Block 1 compared to the “Safe” condition during Block 3, *p* = 0.010, *d* = 1.31. However, with only this significant result, no general trend can be observed regarding task-related MW in both condition. We can only say that task-related MW seems to decrease in the middle of the “Safe” condition, whereas no particular trend can be seen for the “Risky” condition.

**FIGURE 5:**
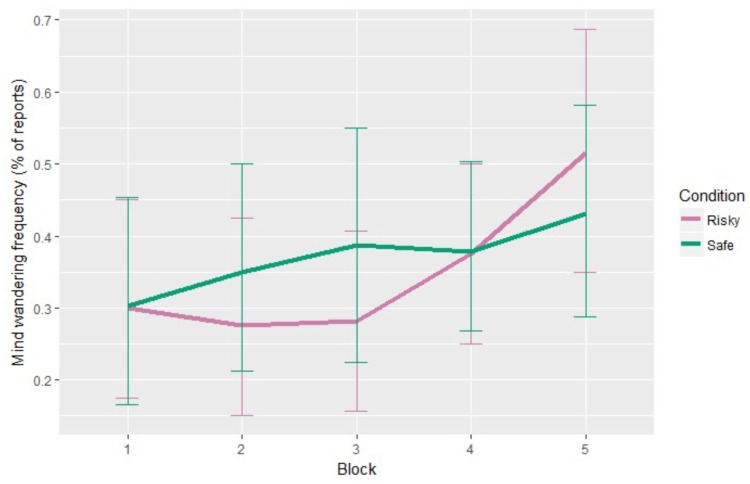
Task-unrelated MW frequency evolution for each condition (error bars show the 95% confidence intervals based on bootstrap).

Task-unrelated MW frequency also changed with time-on-task (see **Figure 5**), χ^2^ = 14.50, *p* = 0.006. Without specific *a priori* predictions regarding the block-by-block evolution, we used *post hoc* tests with *glht* and *mes* functions to uncover the exact evolution of the task-unrelated MW frequency. Tukey’s tests revealed that task-unrelated MW frequencies were significantly higher in Block 5 compared to Block 1, *p* = 0.006, *d* = 0.55, Block 2, *p* = 0.013, *d* = 0.51, and Block 3, *p* = 0.047, *d* = 0.43. This demonstrates a significant increase in the task-unrelated MW frequency toward the end of each session, which is consistent with the existing literature (Krimsky et al., 2017). On the contrary, task-unrelated MW did not show any influence by the condition on its levels, nor on its timely evolution. Given that conditions varied with regard to reliability, thus eliciting different levels of trust (see the following analysis of trust ratings), this result argues against any influence of trust on task-unrelated MW levels.

We continued our analysis by looking at correlations between task-unrelated MW rates, trust and perceived mental demand for each subject. We used the *lme* function to perform a linear mixed-effect analysis, despite a different number of reports between attentional states. We defined a random intercept using “Subjects” and a random slope using “Condition.” Visual inspection of residual plots did not reveal any obvious deviations from normality or homoscedasticity. Each model, starting from the baseline without any predictor, added one predictor or interaction to the preceding model, until the complete model was reached. P-values were obtained by likelihood ratio tests, using ANOVA on nested models. All results are gathered in **Table 3**, bold values being significant.

**Table 3:** Summary of statistics regarding the influence of trust and perceived mental demand over task-unrelated MW frequency.

Effect added	Degrees of freedom	χ^2^	*p*-value
Trust	1	0.017	0.895
Mental demand	2	2.48	0.115

Overall, the analysis of the task-unrelated MW frequency showed that there was no significant interaction between trust ratings nor perceived mental demand ratings with task-unrelated MW frequency. However, task-unrelated MW frequency increased significantly at the end of the session for both conditions.

### Trust

**FIGURE 6:**
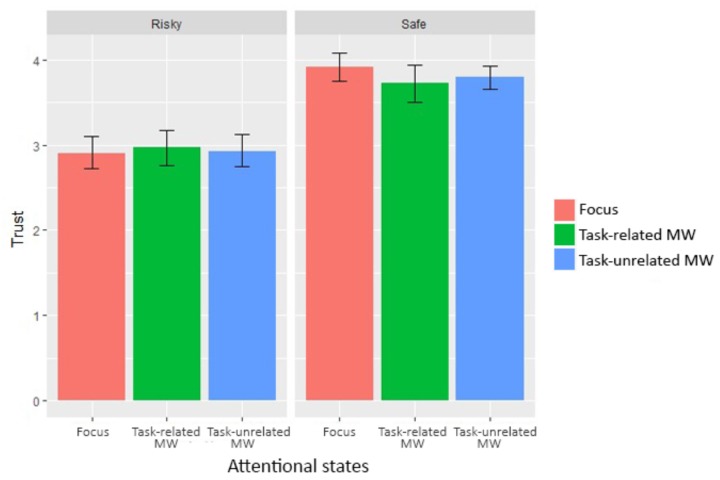
Trust for each condition and attentional state (error bars show the 95% confidence intervals based on bootstrap).

Trust ratings varied substantially between subjects (ranging from 2.12 to 4.58, *M* = 3.38, *SD* = 1.15). We continued to investigate our first hypothesis (influence of trust over MW rates) by looking at the trust evolution between conditions and attentional states. We used the *lme* function to perform a linear mixed-effect analysis, despite a different number of reports between attentional states (see **Figure 6**). We defined a random intercept using “Subjects” and a random slope using “Condition.” Visual inspection of residual plots did not reveal any obvious deviations from normality or homoscedasticity. Each model, starting from the baseline without any predictor, added one predictor or interaction to the preceding model, until the complete model was reached. *P*-values were obtained by likelihood ratio tests, using ANOVA on nested models. All results are gathered in **Table 4**, bold values being significant.

**Table 4:** Summary of statistics regarding the influence of predictors over trust ratings.

Effect added	Degrees of freedom	χ^2^	*p*-value
Condition	1	**14.18**	**<0.001**
Attentional state	3	4.47	0.512
Condition:Attentional state	5	2.09	0.663

*All values in bold are significant at p < 0.05.*

The difference in system reliability significantly impacted trust, since trust ratings reported during the “Risky” condition (*M* = 2.93, *SD* = 1.13) were significantly lower than during the “Safe” condition (*M* = 3.82, *SD* = 0.99), *b* = -0.95, *t*(766) = 4.75, *p* < 0.001. On the contrary, attentional states did not significantly influence trust, χ^2^ = 4.47, *p* = 0.512. In order to determine whether the absence of difference was due to a lack of power, we computed the Type II error using the *pwr* function (Champely, 2017) and *lmer* function (Bates et al., 2017, 4) given that the *lme* function did not provide the necessary information. Computation yielded a Type II error *p* < 0.001, which indicated a very low risk of accepting the null hypothesis, even though there was a significant effect [however, see the critics of *a posteriori* power analysis using the data by Hoenig and Heisey (2001)]. As expected, manipulating system reliability modified trust in the system capabilities. On the contrary, attentional states demonstrated no influence on trust ratings.

### Perceived Mental Demand

Mental demand ratings varied between subjects (ranging from 1.02 to 3.39, *M* = 1.78, *SD* = 0.78). We investigated our second hypothesis (decoupling hypothesis within automated environments) by looking at perceived mental demand evolution between conditions and attentional states. We used the *lme* function to perform a linear mixed-effect analysis including different number of reports between attentional states. We defined a random intercept for subjects and a random slope for condition. This allowed our model to suppress any deviation caused by individual differences and reactions to conditions, thus accounting for repeated measures variables. Visual inspection of residual plots did not reveal any obvious deviations from normality or homoscedasticity. Each model, starting from the baseline without any predictor, added one predictor or interaction to the preceding model, until the complete model was reached. *P*-values were obtained by likelihood ratio tests using ANOVA on nested models. All results are gathered in **Table 5**, bold values being significant.

**Table 5:** Summary of statistics regarding the influence of predictors over perceived mental demand ratings.

Effect added	Degrees of freedom	χ^2^	*p*-value
Condition	1	**5.94**	**0.015**
Attentional state	3	**23.97**	**<0.001**
Condition: Attentional state	5	0.89	0.827

*All values in bold are significant at p < 0.05.*

**FIGURE 7:**
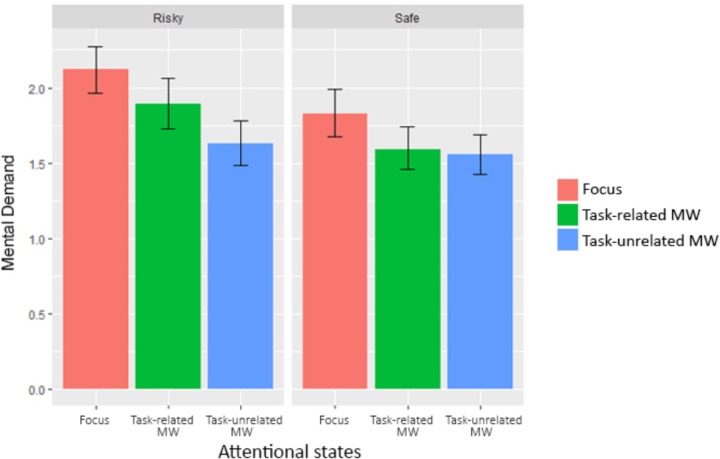
Mental demand for each condition and attentional state (error bars show the 95% confidence intervals based on bootstrap).

The difference in system reliability produced a significant effect on perceived mental demand (see **Figure 7**).

Reported mental demand were significantly lower during “Safe” Condition (*M* = 1.66, *SD* = 0.84) than during “Risky” condition (*M* = 1.88, *SD* = 0.94), *b* = -0.23, *t*(766) = -2.32, *p* = 0.021. Our protocol validate our hypothesis of decrease mental demand when working with higher levels of automation. In addition, we were interested in knowing if all attentional states were different from each other. We used Tukey’s *post hoc* tests to break down the effect. Mental demand reports when focused were significantly higher than those associated with task-related MW, *p* = 0.029, *d* = 0.25, and task-unrelated MW, *p* < 0.001, *d* = 0.43. However, there was only a non-significant tendency for mental demand reports associated with task-unrelated MW to be lower than those associated with task-related MW, *p* = 0.073, *d* = -0.19.

### Oculometry

**FIGURE 8:**
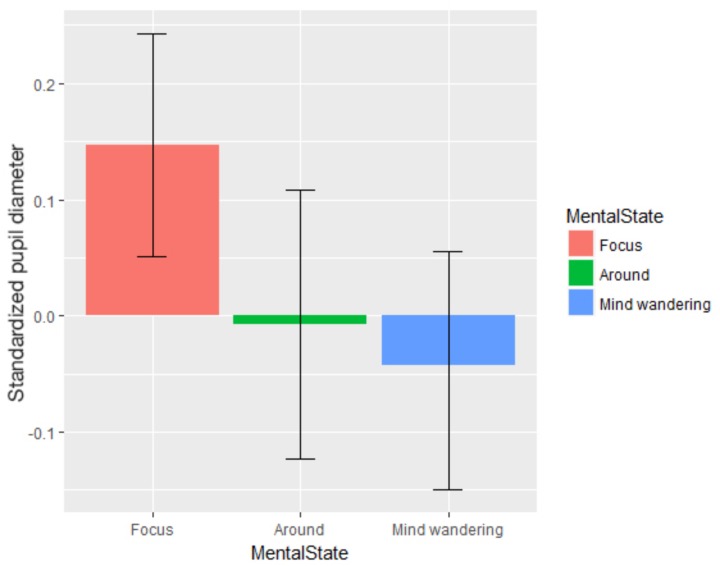
Pupil diameter standardized for each attentional state (error bars show the 95% confidence intervals based on bootstrap).

**FIGURE 9:**
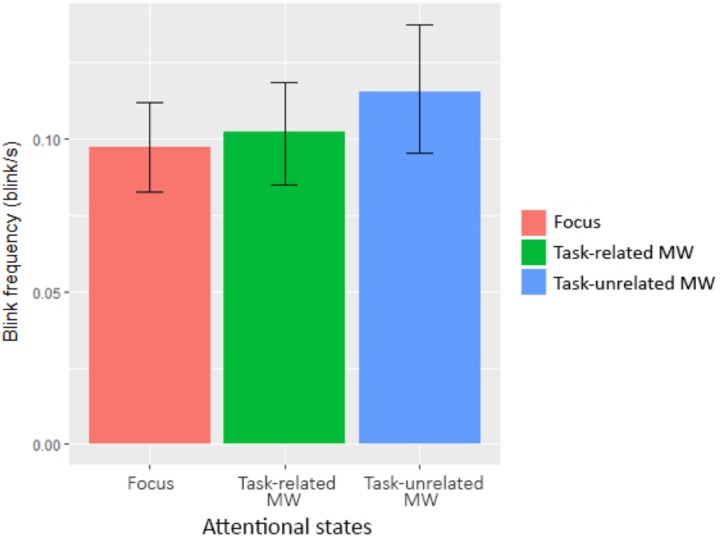
Blink frequency for each attentional state (error bars show the 95% confidence intervals based on bootstrap).

In order to investigate our second hypothesis (decoupling hypothesis within automated environments) from the physiological aspect, we looked at oculometric data through attentional states. After looking at pupil diameter data, we took the 10 s preceding each questionnaire for further analysis. We used the *lme* function to compute a linear mixed-effect analysis despite different number of reports between attentional states. We considered Blocks as a categorical variable. We defined a random intercept for subjects. No random slope was possible because of the convergence problems due to the quantity of data. Visual inspection of residual plots did not reveal any obvious deviations from normality or homoscedasticity. Each model, starting from the baseline without any predictor, added one predictor or interaction to the preceding model, until the complete model was reached. *P*-values were obtained by likelihood ratio tests using ANOVA on nested models. All results are gathered in **Table 6**, bold values being significant. Attentional states showed a significant influence on pupil size, χ^2^(4) = 7.97, *p* = 0.019 (see **Figure 8**). Without specific *a priori* predictions on the evolution of pupil diameter through attentional states, we conducted Tukey’s *post hoc* tests on the model. We saw that pupil diameter when focused was significantly higher than during task-related MW, *p* = 0.036, *d* = 0.08, and task-unrelated MW, *p* = 0.005, *d* = 0.30. On the contrary, blink frequency was significantly higher during task-unrelated MW than when focused, *p* = 0.012, *d* = 0.11 (see **Figure 9**).

**Table 6:** Summary of statistics regarding the influence of time and condition over oculometric markers.

	Focus values		Task-related MW values		Task-unrelated MW values		Attentional state (AS)	
Parameter	*M*	*SD*	*M*	*SD*	*M*	*SD*	χ^2^(2)	*p*-value
Pupil size (mm)	3.93	0.69	3.88	0.62	3.75	0.58	**7.48**	**0.024**
Saccade frequency (sacc/s)	2.25	1.37	2.10	1.11	2.16	1.38	1.74	0.418
Mean fixation duration (s)	0.65	1.28	0.56	0.87	0.56	0.83	0.53	0.767
Blink frequency (blink/s)	0.08	0.09	0.09	0.12	0.11	0.16	**7.77**	**0.021**

*All values in bold are significant at p < 0.05.*

In other words, oculometric measures revealed that pupil diameter decreased and blink rate increased when subjects’ thoughts were distant from the task. On the contrary, no influence of attentional states was observed for saccade frequency and mean fixation duration.

### Performances

In the “Safe” condition, each subject acknowledged on average 133.00 actions of the system (*SD* = 1.32; average of 99% success rate). They also detected on average 23.41 errors (*SD* = 2.74; average of 87% success rate). However, they produced an average of 2.06 false alarms (*SD* = 1.43). In the “Risky” condition, each subject acknowledged on average 132.13 actions of the system (*SD* = 1.77; average of 98% success rate). They also detected on average 4.60 errors (*SD* = 0.63; average of 92% success rate) and produced an average of 1.47 false alarms (*SD* = 2.77).

We investigated the relationship between attentional states and errors. We isolated the attentional reports that included an action of the participant at most 10 s before (6 for the “Risky” condition, 7 for the “Safe” condition). None of these reports included an error for the “Altitude change” command. Therefore, we focused the analysis on the “Acknowledgment” command misses. We used the *lme* function to compute a linear mixed-effect analysis. We defined a random intercept for subjects and a random slope for “Condition.” Visual inspection of residual plots did not reveal any obvious deviations from normality or homoscedasticity. Each model, starting from the baseline without any predictor, added one predictor or interaction to the preceding model, until the complete model was reached. *P*-values were obtained by likelihood ratio tests using ANOVA on nested models. Attentional states showed a significant influence on errors, χ^2^(2) = 8.40, *p* = 0.015. Without specific *a priori* predictions on the evolution of errors through attentional states, we conducted Tukey’s *post hoc* tests on the model. We saw that errors were significantly lower when people thought about task-related matters compared to when they were focused, *p* = 0.013, *d* = -0.61. All other comparisons were not significant. It is possible that thinking too much about matters not directly related to the present decisions (like their performance) makes participants miss some actions. However, this is not compliant with the absence of significant difference between focus and task-unrelated MW. Another more likely possibility could be that participants start thinking about their mistakes for a few seconds after making it, leading them to report task-related MW.

Because the experimental protocol was not designed to answer performance related hypothesis, we will not discuss further the results obtained regarding this point. Instead, the performance measures are given for reproducibility purpose.

## Discussion

We studied the impact of automation reliability on task-unrelated MW frequency and the influence of the MW induced perceptual decoupling on task engagement. Our protocol succeeded in inducing significant differences in trust and perceived workload ratings. Three main results have been shown: (1) task-unrelated MW induced a decoupling from the task which lowered engagement, (2) the perceptual decoupling extended to task-related MW and (3) task-unrelated MW propensity was not linked with trust in the system reliability. We discuss these results below.

The first result is the behavioral and physiological evidence supporting an impact of the task-unrelated MW induced perceptual decoupling on the engagement of operator.

According to the decoupling hypothesis (Schooler et al., 2011), our mind decouples attention from sensory information to sustain prolonged MW. With minimum impact of external information, it becomes dramatically more difficult for operators to perceive and encode external information during task-unrelated MW episodes. We highlighted the effects of this perception decoupling on mental demand, pupil diameter and blink frequency. Firstly, mental demand decreased when participants reported task-unrelated MW. Participants may have experienced a reduced sensitivity to the characteristics of the task and not updated their perceived mental demand. Another possibility is that they answered the probes with limited attention, again relying on information gathered while they were focused. Either way, participants did not spend more cognitive resources on updating their mental model of the situation. This could explain why task-unrelated MW has been shown to disrupt online adjustment of behavior (Kam et al., 2012), since participants might have been operating with an out-of-date model of the situation. Thoughts not directly linked to current task decisions also decreased pupil diameter. This is in line with studies investigating the trade-off between exploration-exploitation (Jepma and Nieuwenhuis, 2011). Indeed, MW is a characterized state of exploitation of information already acquired – e.g., memories – and does not use sensory information except for its ignition point (Seli et al., 2016). Moreover, the literature on vigilance already linked a lower pupil baseline to periods of lower sensibility to external stimuli (Nishiyama et al., 2007; McIntire et al., 2014). It should be noted that some studies highlighted a higher pupil baseline during task-unrelated MW (Smallwood et al., 2011; Franklin et al., 2013). Nevertheless, two recent studies by Unsworth and Robison (2016) and Konishi et al. (2017) observed an inverse U-curve relationship between the pupil diameter and performance. A smaller pupil diameter was linked with a decrease in performances and MW episodes as internally directed cognition. In contrast, a larger pupil diameter was correlated with external distractions (e.g., conversation, noise, or itching) and was also accompanied with a decrease in performance. These studies corroborate our results, while explaining apparent contradictory results. Finally, blink frequency increased during MW episodes. Blinks are known to disrupt visual information processing on two levels: they occlude the retina and they trigger cortical deactivation of the areas responsible for visual information processing (Bristow et al., 2005). Overall, these three measures support the decoupling induced by task-unrelated MW, for both the behavioral and physiological aspects. Far from being anecdotal, the perception of task demands by operators disengaged from the task was found to not be aligned with reality, and these might be unable to perform efficiently. This could lead to automation issues, as described by Parasuraman and Riley (1997). If they had the possibility of performing some tasks or letting the automation handle it, their inaccurate evaluation of the situation may lead them to either choose to handle something manually even though they do not have the cognitive resources for it (disuse), or let the automation do it despite some previous errors (misuse).

Our second result is the extension of the decoupling evidence to the task-related MW. Both mental demand and pupil diameter were significantly lower when participants reported task-related MW compared to being focused. All measures influenced by attentional states – mental demand, pupil diameter and blink frequency – showed the same linear pattern, placing measures linked to task-related MW between those associated with being focused and with task-unrelated MW. Such results are supported by the three-state engagement model of MW (Cheyne et al., 2009). This model proposes three states of MW corresponding to three intensities of decoupling from the task. The model revealed consistent temporal associations between performance and MW levels. The model also revealed bidirectional effects between MW and performance, suggesting that MW can lower performance via the decoupling effect, but also that poor performance can create task-related MW. However, one must remain cautious about the extension of the decoupling hypothesis to MW that includes thoughts related to the task. Blink frequency, which was significantly different between the “Focus” and MW states, was not significantly different between the “Focus” and “Around” states. Further studies are needed to assess the range of thoughts inducing perceptual decoupling, and whether MW episodes indeed possess a depth-modulating perceptual decoupling.

Overall, our results contradict Head and Helton (2016). They argued that participants may rationalize their poor performance by reporting task-related MW; perceptual decoupling causing poor performance would then cause a MW report, and not the other way around, leading researchers to mistake the cause for the consequence. They further strengthen their argument by reporting the results of a GO/NOGO task with inserted words before each stimulus. Their analysis did not show any link between MW and word perception. As detailed in the method, we took into account their results in different aspects of our protocol, to ensure that our experiment would not be taxed with the same flaw. First, the distribution of experience-sampling probes was not correlated with events during the obstacle avoidance task, in order to minimize performance influence over experience-sampling reports. Second, we only kept in our analysis epochs without actions (intervals where participants did not click on any button). Third, we introduced among the attentional probe answers the possibility of reporting “task-related MW,” which we treated separately. Our results are in line with the literature, supporting the decoupling hypothesis for both task-related and task-unrelated MW. Moreover, although some paradigms may indeed be biased by this phenomenological flaw [e.g., oddball or GO/NOGO tasks (Robertson et al., 1997; Braboszcz and Delorme, 2011; Forster and Lavie, 2014)] many others cannot be criticized with the same arguments. Continuous metrics show a similar negative influence of MW on performance (He et al., 2011; Kam et al., 2012; Cowley, 2013; Yanko and Spalek, 2014; Dündar, 2015); if participants were to realize that they did wrong, they would directly correct their behavior. If performance before MW probes were lower, it would mean that participants were not aware of their poor performance. Similarly, many studies highlighted a link between overall performance and the propensity to MW by measuring MW propensity before or after the task; for example, with questionnaires (Galera et al., 2012; Mrazek et al., 2013; Berthié et al., 2015). Therefore, participants could not rationalize their poor performance by reporting MW reports. Nevertheless, all MW researchers should consider this reflection when designing their protocol. Future research should investigate a possible bidirectional link between MW and performance.

Finally, our third result concerns the converging evidence that task-unrelated MW frequency is not linked to trust. Correlation tests did not show any association between task-unrelated MW frequency and trust ratings. Multilevel regression showed no influence of attentional states on trust ratings with significantly low Type II error. Even though we cannot assert that trust is not linked to attentional states, this result supports this hypothesis. A first explanation could be that we failed to highlight the influence of reliability over MW. Complacency may have a dynamic necessitating more time to take place. Operators generally are subjected to thousands of working hours when supervising their system, whereas in this case we tested novices. Investigating experts in similar settings could reveal different results. Another possibility, which could explain the increase in MW frequency for novices when supervising automation (Gouraud et al., 2017b), would be the impact of a loss of agency. Agency is the feeling of control produced by the idea that our actions are producing the observed effect. Obhi and Hall (2011) highlighted a decrease in one’s feeling of agency in automated environments, compared to the same task done manually. Knowing that a decrease in the feeling of agency leads to the operator’s disengagement from the task (Haggard, 2017), human operators might disengage from the task and allocate a lower amount of cognitive resources to the task. Resources could then be used for task-unrelated MW maintenance. This hypothesis is tightly linked with motivation and the Self-Determination Theory (Ryan and Deci, 2000; Szalma, 2014). Even though participants were volunteers, the task proposed was purposely boring and did not produce much motivation. The inability of the task to support autonomous behavior and internalization of the goal may lower motivation and create an externalization of task goals – i.e., a process by which operators reject the intrinsic value of a goal. Ultimately, participants could voluntarily redirect their attention and cognitive resources whenever possible toward more personally interesting and useful matters, increasing MW frequency. Task-unrelated MW would act both as a way to cope with boredom (Cummings et al., 2015) and a process to optimize time and resources (Gouraud et al., 2017a). Further studies building on agency and task-unrelated MW literatures should investigate this hypothetical link.

In the near future, the massive use of automation within many different fields will reinforce the problem of perceptual decoupling induced by MW. Even though training can help to mitigate this phenomenon, extensive research on better automation and human-system interfaces in needed to cope with this problem (Hancock, 2013). Adaptive automation has been studied for a few decades and shows promising results (Kaber and Endsley, 2004; Abbass et al., 2014; Berberian et al., 2017). Adaptive automation adapts the level of automation according to one or multiple physiological measures, in order to mitigate OOTL effects. To integrate efficient adaptive automation within safety-critical environments, system designers need to understand the ways in which variables affect vigilance, trust, mental demand and other abilities necessary for efficient control.

## Author Contributions

All authors listed have made a substantial, direct and intellectual contribution to the work, and approved it for publication.

## Conflict of Interest Statement

The authors declare that the research was conducted in the absence of any commercial or financial relationships that could be construed as a potential conflict of interest. The reviewer DR and handling Editor declared their shared affiliation.
